# Sex Difference in Global Burden of Major Depressive Disorder: Findings From the Global Burden of Disease Study 2019

**DOI:** 10.3389/fpsyt.2022.789305

**Published:** 2022-02-21

**Authors:** Sangzi Li, Yufeng Xu, Leilei Zheng, Hu Pang, Qianni Zhang, Lixia Lou, Xingru Huang

**Affiliations:** ^1^School of Marxism, Zhejiang University, Hangzhou, China; ^2^School of Design and Fashion, Zhejiang University of Science and Technology, Hangzhou, China; ^3^Department of Ophthalmology, The Second Affiliated Hospital of Zhejiang University, College of Medicine, Hangzhou, China; ^4^Department of Psychiatry, The Second Affiliated Hospital of Zhejiang University, College of Medicine, Hangzhou, China; ^5^School of Electronic Engineering and Computer Science, Queen Mary University of London, London, United Kingdom

**Keywords:** major depressive disorder, sex difference, Global Burden of Disease Study, disability-adjusted life-year, human development index

## Abstract

**Objectives:**

Globally, major depressive disorder (MDD) is considered to be a leading cause of disability. In this article, we aim to investigate the sex difference in global burden of MDD by year, age, and socioeconomic development, utilizing disability-adjusted life-years (DALYs).

**Methods:**

Global and national sex-specific DALY estimates caused by MDD from 1990 to 2019 and in different age groups were obtained from the Global Burden of Disease (GBD) Study 2019. Human development index (HDI) was used as an indicator of national socioeconomic development. Spearman correlation and linear regression analyses were performed to explore the relationship between national socioeconomic development and sex difference in MDD burden.

**Results:**

Sex difference in global burden of MDD persisted between 1990 and 2019, with age-standardized DALY rates being 352 among males vs. 593 among females in 1990 and 354 vs. 564 in 2019. Females had higher burden of MDD than males at the same age. Disability-adjusted life-years numbers and rates among both sexes rapidly increased with age for those aged 10–24 years, along with gradually enlarging sex difference. Age-standardized DALY rates among females were higher than that among males for each HDI-based country group (*P* < 0.001). National age-standardized DALY rates among both sexes were negatively related to HDI. However, female-to-male age-standardized DALY rate ratios were positively associated with HDI (Spearman *r* = 0.383, *P* < 0.001; standardized β = 0.300, *P* < 0.001).

**Conclusion:**

Although some improvement in sex difference in global burden of MDD has been achieved, it still persists in the past three decades, with females bearing more burden than males. To reduce sex difference in global MDD burden, more attention should be paid to young people and people in developed countries. The findings highlight the importance of making sex-specific health policy to manage mental impairment caused by MDD.

## Introduction

Depression is a common mental disorder affecting approximately 300 million people of all ages worldwide (1). It is a leading cause of disability and contributes greatly to the Global Burden of Disease (GBD). The effects of depression can be long-lasting or recurrent and can dramatically affect a person's ability to function and to live a rewarding life. The GBD study has estimated health burden of depression using disability-adjusted life-years (DALYs). In 2019, the number of DALYs caused by depression accounted for 1.85% of all DALYs worldwide, with major depressive disorder (MDD) accounting for 1.47% and dysthymia for 0.38% (2). Major depressive disorder is an episodic mood disorder with shorter-lasting but more severe symptoms than dysthymia. Females are more likely to suffer from MDD than males, with global prevalence rate being 3.0% in females and 1.8% in males (2). Several factors such as biological, affective, cognitive, and sociocultural factors have been suggested to contribute to female vulnerability to MDD (3, 4).

Based on DALY data for depressive disorders from the GBD study 2015, significant correlations between gender disparity in depressive disorders and diverse aspects of social inequality (including gender inequality and wealth inequality) have been detected (5). Disability-adjusted life-year data allow direct comparisons of MDD burden by sex from multiple perspectives. In our previous studies, DALY data had been used to demonstrate the global sex disparity in other non-communicable diseases from the aspects of year, age, and socioeconomic status (6–8). Despite the fact that MDD is treatable under psychological and pharmacologic therapies, sex disparity in MDD burden remains a major concern for policymakers focusing on prevention and control of mental disorders. The general patterns of sex difference in MDD burden will be of significance for making sex-specific health policy to combat MDD. Therefore, the purpose of this study is to compare multiple aspects of sex difference in global burden of MDD by year, age, and socioeconomic status, using the most up-to-date DALY estimates from the GBD study 2019 (2).

## Materials and Methods

### Sex-Specific Burden of MDD

Major depressive disorder is defined according to the diagnostic criteria of the *International Classification of Diseases* or the *Diagnostic and Statistical Manual of Mental Disorders* (9, 10). Detailed calculation methods of DALY estimates have been previously described in the GBD study 2019 (2). Disability-adjusted life years are the sum of years of life lived with disability (YLDs) and years of life lost due to premature death. As MDD is a non-fatal disease, DALYs are equivalent to YLDs. Disability-adjusted life years caused by MDD are extracted from the Global Health Data Exchange, a catalog of global health and demographic data that allows directly downloading the GBD study data (11). The following data are extracted: (1) global sex-specific DALY numbers, crude rates (DALYs per 100,000 population), and age-standardized rates (age-standardized DALYs per 100,000 population) from 1990 to 2019; (2) World Health Organization (WHO) regional sex-specific age-standardized DALY rates from 1990 to 2019; (3) global sex-specific DALY numbers and crude rates in different age groups in 2019; and (4) national sex-specific age-standardized DALY rates in 2019.

### National Socioeconomic Development

Human development index (HDI) is a composite index developed by the United Nations to evaluate the socioeconomic development of countries around the world (12). Human development index considers three dimensions of human development, namely, life expectancy, education, and per-capita income. Human development index data in 2019 were extracted from the Human Development Report 2020 (12). The values of HDI range from 0 to 1, with higher HDI indicating higher level of national socioeconomic development. Countries were classified into four groups by HDI, including low-HDI countries (0 < HDI <0.554), medium-HDI countries (0.554 ≤ HDI <0.703), high-HDI countries (0.703 ≤ HDI <0.804), and very-high-HDI countries (0.804 ≤ HDI <1) (12).

### Statistical Analyses

Globally, national age-standardized DALY rates among female population and among male population were compared using Wilcoxon signed ranks test. Such comparisons were also performed for each HDI-based country group. Spearman correlation analyses and linear regression analyses were conducted to assess the association of national age-standardized DALY rates among female population, age-standardized DALY rates among male population, and female-to-male age-standardized DALY rate ratios with HDI. SPSS 23 (IBM, Chicago, IL, USA) was used for statistical analyses, and *P* < 0.05 was considered statistically significant.

## Results

### Sex Difference in MDD Burden by Year

**Figure 1:**
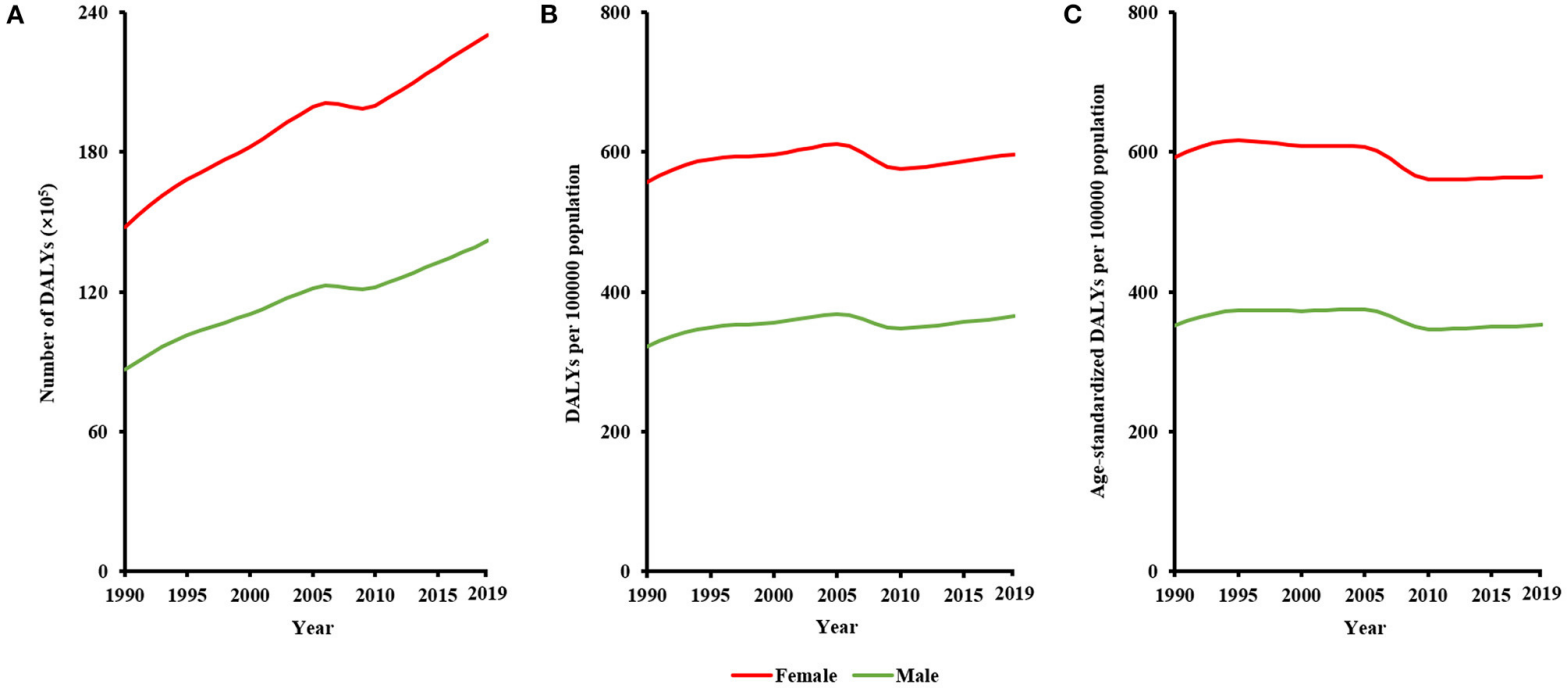
Sex difference in global burden of major depressive disorder from 1990 to 2019, in terms of **(A)** DALY numbers, **(B)** crude DALY rates, and **(C)** age-standardized DALY rates.

**Figure 2:**
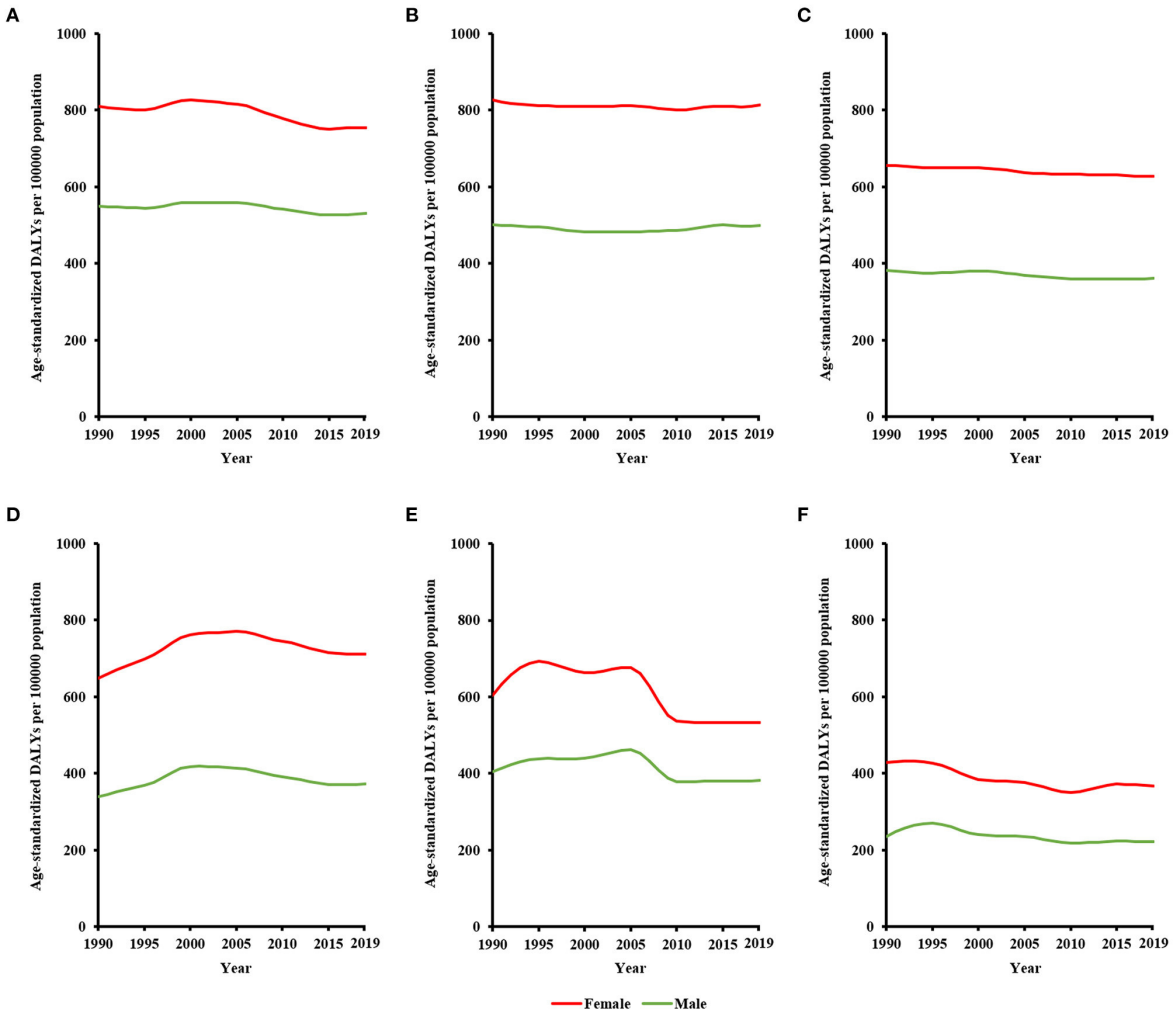
Sex difference in WHO regional burden of major depressive disorder from 1990 to 2019, in terms of age-standardized DALY rates: **(A)** African region, **(B)** Eastern Mediterranean region, **(C)** European region, **(D)** region of the Americas, **(E)** Southeast Asia region, and **(F)** Western Pacific region.

Sex difference in global burden of MDD persisted between 1990 and 2019 (Figure 1). Sex difference in terms of DALY numbers continuously increased during the past three decades, with the difference being 6,127,600 (8,666,840 among male population vs. 14,794,440 among female population) in 1990 and 8,836,198 (14,183,272 among male population vs. 23,019,470 among female population) in 2019. After controlling for population quantity, sex difference in crude DALY rates changed slightly, with the difference being 235 (322 among male population vs. 557 among female population) in 1990 and 231 (366 among male population vs. 597 among female population) in 2019. After controlling for both population quantity and age structure, sex difference in age-standardized DALY rates continuously decreased, with the difference being 241 (352 among male population vs. 593 among female population) in 1990 and being 210 (354 among male population vs. 564 among female population) in 2019. Sex difference in MDD burden persisted in all WHO regions from 1990 to 2019 (Figure 2). Sex difference in terms of age-standardized DALY rates was largest in the region of the Americas, followed by Eastern Mediterranean Region, and was smallest in the Western Pacific Region.

### Sex Difference in MDD Burden by Age

**Figure 3:**
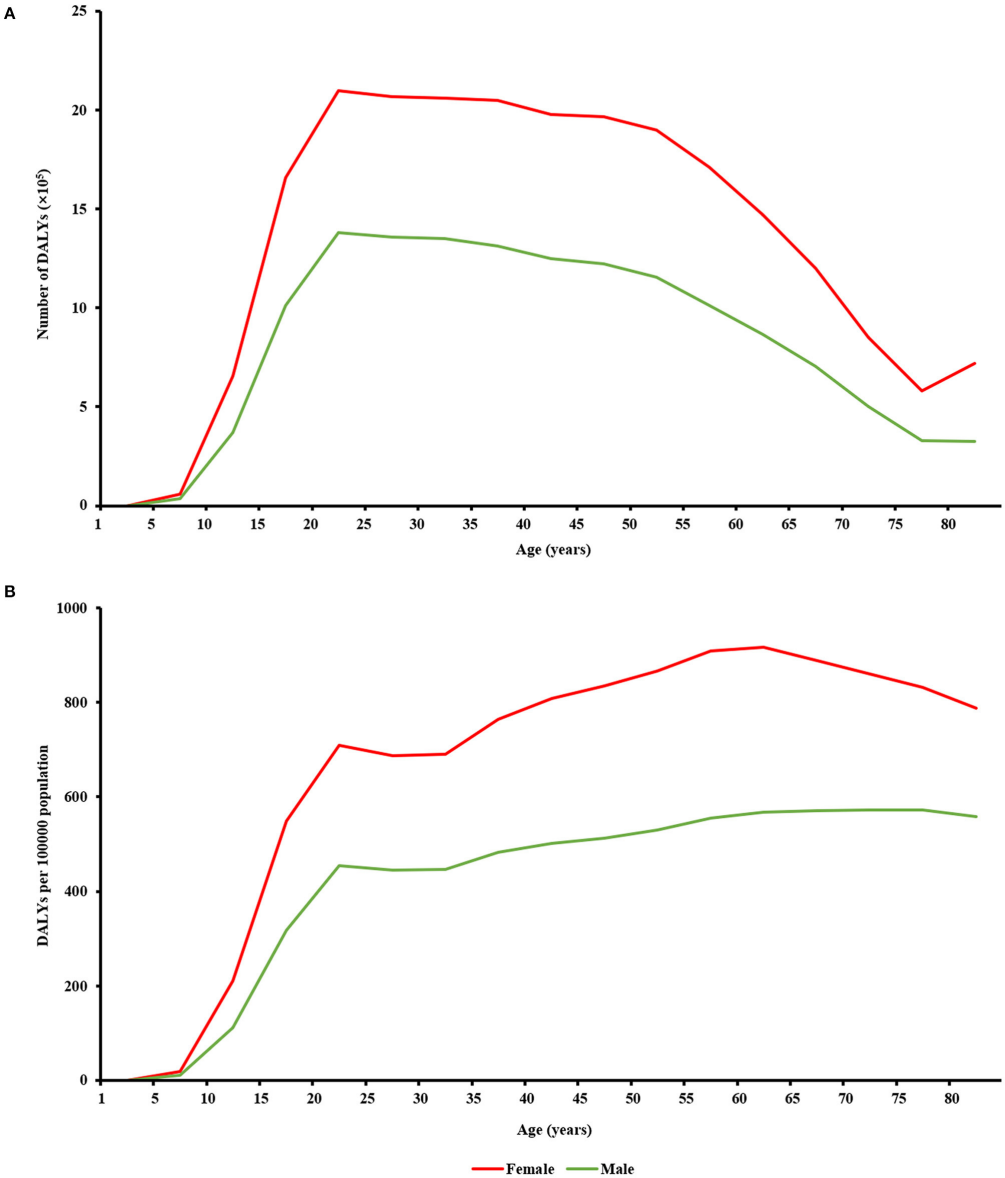
Sex difference in global burden of major depressive disorder in different age groups in 2019, in terms of **(A)** DALY numbers and **(B)** crude DALY rates.

Globally in 2019, female population had higher burden of MDD than male population at the same age (Figure 3). Disability-adjusted life-year numbers and DALY rates among both sexes rapidly increased with age for those aged 10–24 years, along with gradually enlarging sex difference. In the 20- to 24-year age group, sex difference in DALY numbers was 716,182 (1,380,868 among male population vs. 2,097,050 among female population), and sex difference in DALY rates was 255 (454 among male population vs. 709 among female population). Sex difference in DALY numbers was greatest (being 746,586) in the 50- to 54-year age group and decreased with age for those aged 55–79 years. Sex difference in DALY rates was greatest (being 353) in the 55- to 59-year age group and decreased with age for those 60 years or older.

### Sex Difference in MDD Burden by Socioeconomic Development

**Figure 4:**
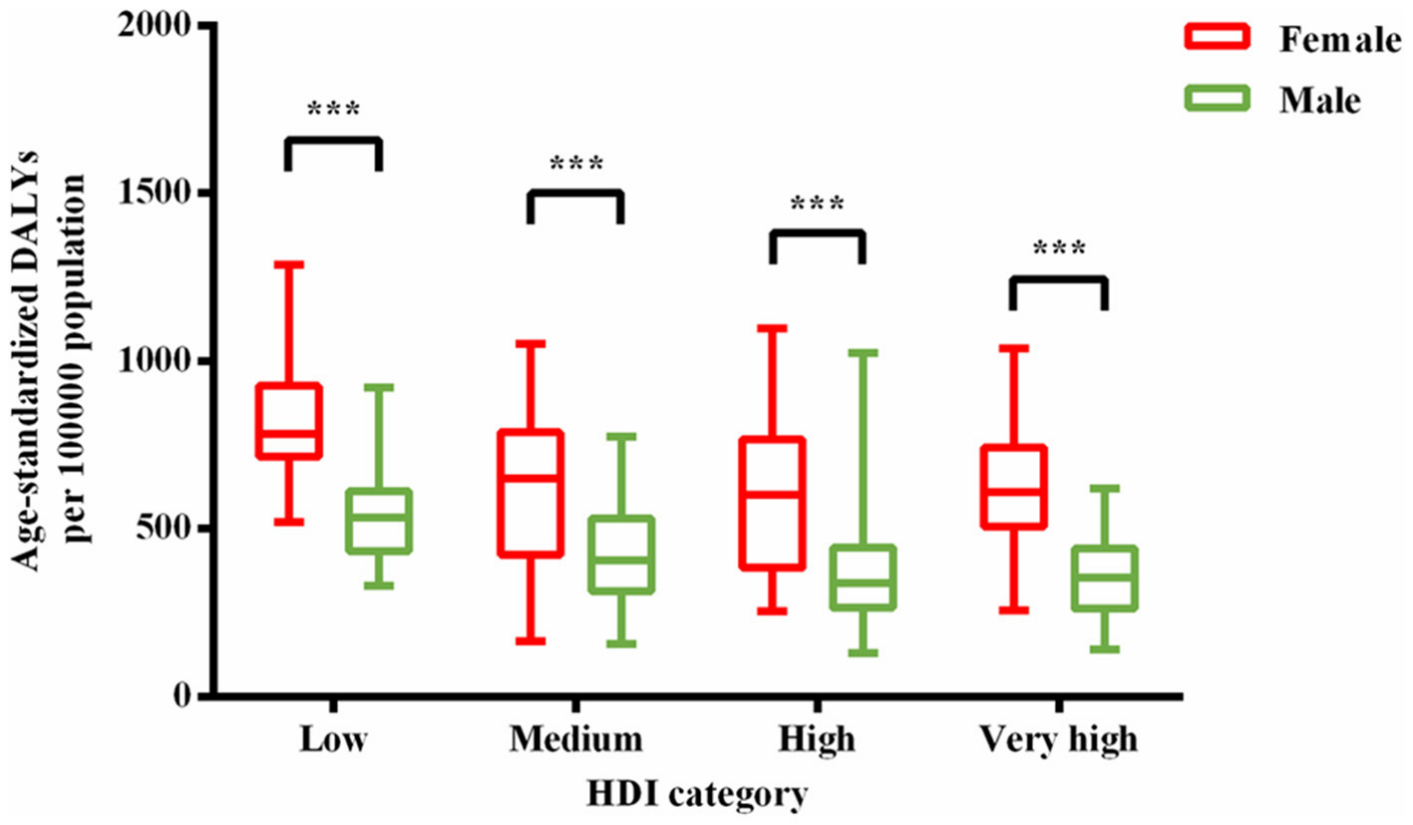
Age-standardized DALY rates among female population were higher than that among male population for each HDI-based country group. Lines inside the boxes indicate the medians; boxes, the 25th and 75th percentiles; and lines outside the boxes, the minimum and the maximum. ***P < 0.001.

**Figure 5:**
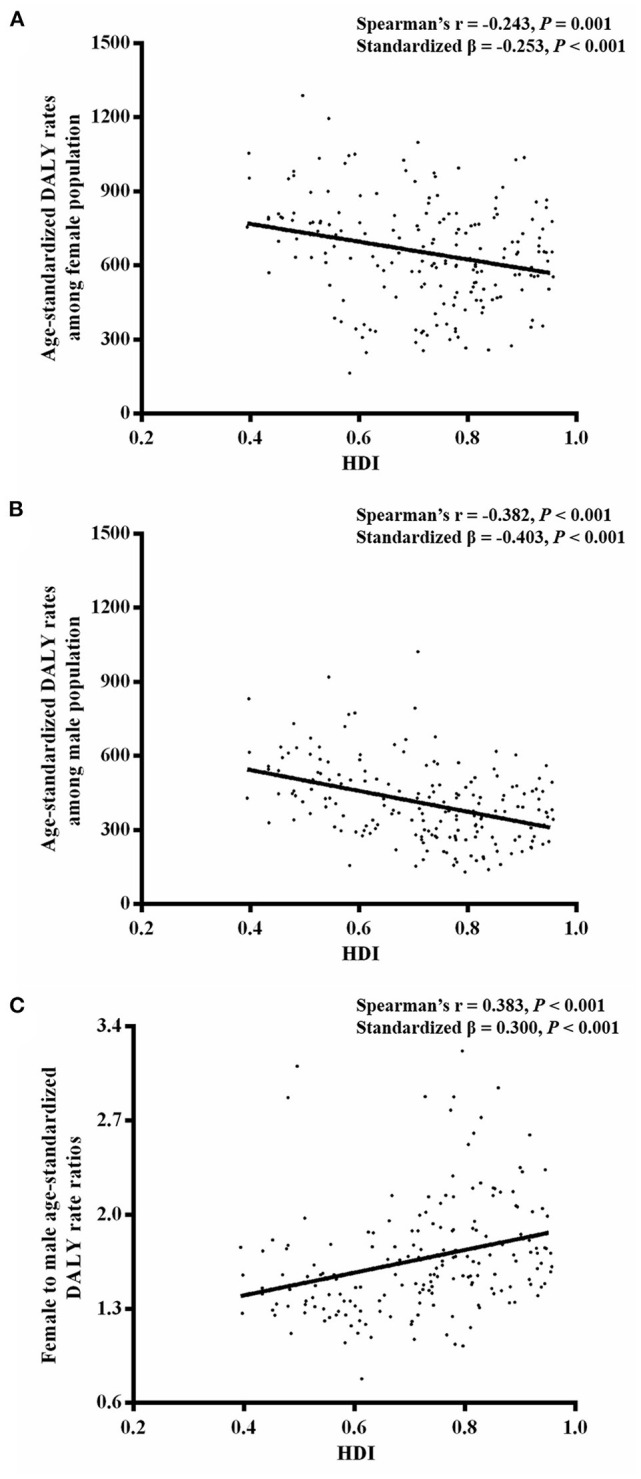
Linear relationship **(A)** between national age-standardized DALY rates among female population and HDI, **(B)** between national age-standardized DALY rates among male population and HDI, and **(C)** between national female-to-male age-standardized DALY rate ratios and HDI.

Wilcoxon test showed that for 204 countries included in the GBD study 2019, age-standardized DALY rates caused by MDD among female population (median [interquartile range]: 642 [476–782]) were significantly greater than that among male population (378 [295–293]) (*Z* = −12.367, *P* < 0.001). Human development index data were available for 187 countries, including 64 very-high-HDI countries, 53 high-HDI countries, 37 medium-HDI countries, and 33 low-HDI countries. Age-standardized DALY rates among female population were higher than that among male population for very-high-HDI (females vs. males: 611 [507–744] vs. 356 [262–442]; *Z* = −6.955, *P* < 0.001), high-HDI (602 [384–768] vs. 339 [265–445]; *Z* = −6.334, *P* < 0.001), medium-HDI (651 [423–788] vs. 407 [315–532]; *Z* = −5.197, *P* < 0.001), and low-HDI (784 [716–926] vs. 534 [434–614]; *Z* = −5.012, *P* < 0.001) countries (Figure 4). National age-standardized DALY rates among female population (Spearman *r* = −0.243, *P* = 0.001; standardized β = −0.253, *P* < 0.001), as well as age-standardized DALY rates among male population (Spearman *r* = −0.382, *P* < 0.001; standardized β = −0.403, *P* < 0.001), were negatively related to HDI (Figure 5). However, female-to-male age-standardized DALY rate ratios were positively associated with HDI (Spearman *r* = 0.383, *P* < 0.001; standardized β = 0.300, *P* < 0.001).

## Discussion

This study revealed that sex difference in global burden of MDD had persisted since 1990, with only a small improvement achieved. Globally, female population had higher burden of MDD than male population at the same age. Major depressive disorder burden rapidly increased with age for those aged 10–24 years, along with gradually enlarging sex difference. Despite less MDD burden in more developed countries, greater sex difference was found in these countries.

According to the data of GBD study 2019, global age-standardized prevalence rates of MDD decreased from 2,357 per 100,000 population in 1990 to 2,286 per 100,000 population in 2019 (11). However, the global health improvement of MDD did not mean much smaller sex difference. Higher burden of MDD among females had persisted for decades in all regions of the world. Biological features could explain part of the sex difference (4). The heritability of MDD is estimated at 30–40% (13), with mixed evidence for a stronger genetic risk for females than for males (13–15). Specifically, females show higher levels of inflammatory, neurotrophic, and serotonergic markers and a stronger relationship between levels of some inflammatory and neurotrophic markers and the severity of depressive symptoms (16). In addition, females are more prone to disparities in employment, salary rates, education, and psychological stress, which are probably important environmental predictors of depression (4, 17, 18). Findings from the WHO World Mental Health Surveys revealed a temporal trend of a converging gender gap in depressive disorders in countries with less traditional gender roles (19). Thus, monitoring of changing social and cultural trends in environment risk factors possibly leading to the gender gap is of particular importance.

The onset of MDD can occur at any stage of life. Our study shows that burden of MDD rapidly increased with age in young people, along with gradually enlarging sex difference, suggesting that adolescence may be a critical time for studying vulnerability to depression. A developmentally sensitive, elaborated cognitive vulnerability-transactional stress model of depression had been proposed to explain the development of sex difference in depression during adolescence (20). Persistent depression is related to serious complications, including poor school performance, recurring depression in adult, and even suicide (21). According to a WHO report, suicide was the second leading cause of death among 15- to 29-year-olds, and close to 800,000 people die due to suicide every year (22). Thus, effective treatment of depression in young people is an important means to reduce the global burden of MDD (21). Our study reveals that sex difference in MDD burden persisted into late life. Multiple predictors, such as negative life events, coping styles, and interpersonal orientation, might account for the sex difference in the elderly in complex ways (23). Elucidating the interactions between the predictors and the life span development of sex difference in MDD would probably contribute to the timely prevention, diagnosis, and treatment of depression in old people.

Using crude DALY rates from the GBD study 2015, Yu (5) found a direct positive relationship between gross domestic product and log-transformed ratio of depressive disorder rates for female to male, after adjusting for regional effects and other socioeconomic factors (such as gender inequality index, Gini index) for 122 countries, which was consistent with our findings. In the current study, we have gone farther by using age-standardized DALY rates (which controls for both population size and composition) and HDI (which is a composite measure of health, education, and income) for 187 countries worldwide. Our findings suggest that higher level of a country's overall development was correlated with greater sex difference in MDD burden. In more developed countries, females' lives are less predetermined, and more choice might mean more conflicts between possible roles, which could lead to greater sex difference in depression (24). However, improving the overall development of a country might indeed reduce the burden of MDD in a specific population (25, 26). It is consistent with the findings of this study that both sexes in more developed countries had less MDD burden. A meta-analysis on socioeconomic inequalities in depression found that each additional year of education led to a 3% decrease in the log odds ratio of being depressed, and a 1% increase in relative ranking on income led to a 0.74% decrease in the log odds ratio of being depressed (27). From the perspective of sex-specific age-standardized DALY rates caused by MDD, more attention should be paid to less developed countries in which both sexes suffered from more MDD burden (28, 29). There exists a vicious cycle of poverty, depression, and disability (30–32). Financial poverty alleviation interventions, such as conditional cash transfer (33, 34) and asset promotion programs (35), have shown some mental health benefits (36). Unfortunately, there lacks common recognition of the signs or symptoms that something may be wrong, nor of the fact that depression is a treatable illness. Only a minority of people with MDD received minimally adequate treatment: one in five people in high-income and one in 27 in low-/lower-middle-income countries (37). Therefore, raising public awareness of mental health issues, for instance, by community education and outreach, is important for scaling up care for MDD (38).

This study was susceptible to the limitations of the GBD study 2019, such as data sources and statistical assumption, as mentioned in the GBD report (2). Because of the use of national data rather than provincial or state data, bias may be triggered by geographic variations in DALY estimates. Despite the global view of sex difference in MDD burden presented in this study, the findings would not hold up to a specific district. As GBD data are updated annually, sex difference in global MDD burden during a longer period, as a reflection of disease control effect, could be explored further.

In conclusion, this study demonstrated that sex difference in global MDD burden had persisted for decades, with only small improvement achieved. Females always had higher burden of MDD than males. Sex difference gradually increased with age for young people and was greater in more developed countries. The findings of this study call for more attention on the female vulnerability to MDD and may have important implications for global and national strategies to prevent and control MDD, especially to reduce sex difference in MDD. Although MDD is treatable with therapy, medication, and lifestyle changes, sex difference remains a major concern for managing mental impairment caused by MDD. This study highlights the importance of making sex-specific health policy to reduce global burden of MDD.

## Data Availability Statement

Publicly available datasets were analyzed in this study. This data can be found here: http://ghdx.healthdata.org/gbd-results-tool.

## Author Contributions

SL and YX performed statistical analyses and wrote the manuscript. LZ contributed to the design of the study and critically evaluated the manuscript. HP and LL contributed to the conception and design of the study. QZ critically revised the article. XH participated in the interpretation of the results and critical revision of the article. All authors contributed to the article and approved the submitted version.

## Funding

This work was supported by the National Natural Science Foundation of China (No. 82000948).

## Conflict of Interest

The authors declare that the research was conducted in the absence of any commercial or financial relationships that could be construed as a potential conflict of interest.

## Publisher's Note

All claims expressed in this article are solely those of the authors and do not necessarily represent those of their affiliated organizations, or those of the publisher, the editors and the reviewers. Any product that may be evaluated in this article, or claim that may be made by its manufacturer, is not guaranteed or endorsed by the publisher.
